# The rise of medical training in Portuguese speaking African countries

**DOI:** 10.1186/1478-4491-12-63

**Published:** 2014-11-03

**Authors:** Inês Fronteira, Mohsin Sidat, Mário Fresta, Maria do Rosário Sambo, Celso Belo, Cezaltina Kahuli, Maria Alexandra Rodrigues, Paulo Ferrinho

**Affiliations:** International Public Health and Biostatics Unit, WHO Collaborating Center for Health Policy and Workforce Planning, Instituto de Higiene e Medicina Tropical, Universidade Nova de Lisboa, Lisbon, Portugal; Faculty of Medicine, Universidade Eduardo Modlane, Maputo, Mozambique; CEDUMED, Universidade Agostinho Neto, Luanda, Angola; Faculty of Medicine, Universidade de Katyavala Bwila, Benguela, Angola; Faculty of Health Sciences, Universidade do Lúrio, Maputo, Mozambique; Huambo Faculty of Medicine, Universidade José Eduardo dos Santos, Luanda, Angola; Faculty of Health Sciences, Universidade de Zambeze, Maputo, Mozambique

## Abstract

**Background:**

Medical training has shown to be strategic for strengthening health systems, especially in those countries identified to have critical shortage of human resources for health. In the past few years, several studies have been conducted to characterize and identify major challenges faced by medical schools worldwide, and particularly in Africa. Nevertheless, none has previously addressed medical training issues in Portuguese Speaking African Countries (PSAC). The aim of this study was to establish baseline knowledge of the PSAC’s medical schools in terms of creation and ownership, programmes offered, applicants and registered students, barriers to increased intake of students, teaching workforce and available resources.

**Methods:**

A quantitative, observational, multicentric, cross-sectional study of all medical schools active in 2012 in the PSAC. An adapted version of the questionnaires developed by Chen *et al*. (2012) was sent to all medical schools electronically. Data were analyzed using descriptive statistics.

**Results:**

A total of nine medical schools answered the questionnaire (three from Angola, two from Guinea Bissau and four from Mozambique). Since 2006 an effort has been made to increase the number of medical trainees. Besides the medical degree offered by all schools, some offered other undergraduate and postgraduate training programmes. The number of applicants to medical schools largely outnumbers the available vacancies in all countries but insufficient infrastructures and lack of teaching personnel are important constraints to increase vacancies. The teaching personnel are mainly trained abroad, employed part-time by the medical school and do not have a PhD qualification.

**Conclusion:**

Governments in the PSAC have significantly invested in training to address medical shortages. However, medical schools are still struggling to give an adequate and effective response. Developing a local postgraduate training capacity for doctors might be an important strategy to help retain medical doctors in the home country and develop local faculty capacity.

## Background

A 2011 paper on 168 medical faculties in Africa covered only one in the Lusophone countries of the continent [1, 2]. This paper extends the study to all Portuguese Speaking African Countries (PSAC) with active medical schools in 2012, namely: Angola, Guinea Bissau and Mozambique. Two other PSAC, such as Cape Verde and Sao Tome and Principe, have no medical schools, although Cape Verde is currently engaged in reflecting on the relevance and viability of establishing a local medical training institution [3].

Medical training assumes a strategic role in the effort to correct the major deficit of medical doctors in Africa [1, 2, 4]. The 2006 World Health Report and the Joint Learning Initiative cast light on those shortages, identifying 57 countries with critical shortages. Angola, Guinea Bissau and Mozambique are among the critical shortage countries [5–7].

This shortage was in part the result of the limited number of medical schools and of students enrolled and graduating from local medical programmes, along with a weak knowledge of medical schools’ capacities, strengths and weaknesses [1, 2, 4, 7]. This is further aggravated by attrition of the medical workforce either by emigration, frequently seeking for postgraduate study opportunities abroad, or by moving from the public to the private sector [8–12].

The roadmap drawn to scale up the health workforce for Africa [7] and national policies of several countries in this region, including the PSAC, have acknowledged the need to train more doctors and addressed this need by allowing more medical schools to open and/or those schools already functioning to admit more students. In Angola, Guinea Bissau and Mozambique this expansionist policy has been part of national health and higher education policies and strategies, which has not always been the case in other critical shortage countries: of the 46 countries of WHO’s African Region, 24 do not have a national human resources for health (HRH) policy [5]. If not properly articulated with the strategic planning of the country’s workforce, the expansion of medical education may have deleterious effects, as observed in the Democratic Republic of Congo, where new medical schools were created without accounting for quality of training or the market’s capacity to absorb newly graduates, leading to the closure, early in 2013, of 75 medical schools and departments due to substandard facilities and poor training of medical personnel [13].

The aim of this study was to establish baseline knowledge on the PSACs’ medical schools in terms of creation and ownership, programmes offered, applicants and registered students, barriers to increased intake of students, teaching workforce and available resources. For the purpose of this paper we define medical school as institutions that provide undergraduate training of medical doctors.

## Material and methods

This was a quantitative, observational, multicentric, cross-sectional study. The population of the study comprised all medical schools (private and public) of Angola (n = 8), Guinea Bissau (n = 2) and Mozambique (n = 5), in a total of 17 (Table 1). In Mozambique only five medical schools were included in the study; two private faculties were excluded as they were not legalized - one in Pemba, with only first year students at the time of the study and the other one in Maxixe, without any students. No sample was drawn. Further to the medical schools, the study includes also data on CEDUMED - Centre for Medical Education - an autonomous (since 2012, although established in 2003 within the Faculty of Medicine) academic unit of the University Agostinho Neto (UAN) in Luanda, Angola.

**Table 1:** **University medical faculties in Angola, Guinea Bissau and Mozambique**

Country	Faculty’s university	City	Date of establishment	Ownership (public/private)	Response to the questionnaire
Angola	Agostinho Neto	Luanda	1963	Public (Ministry of Higher Education)	No response
	Jean Piaget^a^	Luanda	2000	Private	No response
	Instituto Superior Técnico Militar^b^	Luanda	2008	Public (Defense Force of Angola)	No response
	Katiavala Bwila	Benguela	2008	Public (Ministry of Higher Education)	Responded
	11 de Novembro	Cabinda	2008	Public (Ministry of Higher Education)	Responded
	Lueji A’Nkonde	Malange	2009	Public (Ministry of Higher Education)	No response
	José Eduardo dos Santos	Huambo	2009	Public (Ministry of Higher Education)	Responded
	Mandume ya Ndemufayo	Lubango	2009	Public (Ministry of Higher Education)	No response
Guinea Bissau	ISCM-CH Universidad de La Habana - Raul Diaz Arguelles	Bissau	1987	^c^	Responded
	Jean Piaget	Bissau	2011	Private	Responded
Mozambique	UniZambeze	Cidade da Beira	2009	Public (Ministry of Higher Education)	Responded
	Eduardo Mondlane	Maputo	1963	Public (Ministry of Higher Education)	Responded
	UniLurio	Nampula	2007	Public (Ministry of Higher Education)	Responded
	Catolica de Moçambique	Cidade da Beira	2000	Private	Responded
	Instituto Superior de Ciências e Tecnologia de Moçambique	Maputo	?	Private	No response

^a^Medical training takes place within a Department of Health Sciences of this University.

^b^Medical training takes place within a Department of Biotechnology and Health Sciences of this University Institute.

^c^The status of this faculty in unclear and has been associated with the School of Public Health, with the public university, with the Ministry of Public Health and with the Ministry of Education.

The medical schools as well as the directors were identified based on local stakeholders and privileged contacts of the authors.

We used an adapted Portuguese version of the questionnaire developed by Chen *et al*. [1]. Electronic questionnaires were sent in Word 97 (Microsoft, Redmond, WA, USA) format by e-mail to the directors of the medical schools in the 3 countries in the second semester of 2012. The directors were asked to fill the questionnaire and return it via e-mail to the research team. All e-mails were sent with a request for receipt of notification. In case of failure to respond until the deadline, a reminder was sent setting another deadline. Two reminders were sent, the last one being in January 2013. Non-respondents were successfully contacted telephonically before assuming non-response.

Questionnaire data were complemented with data collected for non-respondents and respondents from institutional sites, annual reports, literature review and contacts with key-informers other than the directors of the faculties.

Data were entered in a SPSS v.20 database (SPSS Inc., Chicago, IL, USA) and descriptive statistics (counts, relative frequencies, mean and standard deviation and medians) computed. In April 2013 a meeting was held in Lisbon with the medical schools’ directors of Angola and Mozambique where the study results were presented and discussed. After this meeting a second data collection period took place to collect, into a standardized Excel sheet, longitudinal information on vacancies, applications and registrations in the medical programme as well as on the evolution of the composition and skills of the teaching staff.

## Results

The overall response rate was 60%. The response rate in Angola was 37.5% (3 out of 8), 100% in Guinea Bissau (2 out of 2) and 80% in Mozambique (4 out of 5) (Table 1).

### Creation and ownership

Two of the medical schools, one in Maputo, Mozambique and the second in Luanda, Angola, were established in 1963, during the colonial administration. A third medical School was established with Cuban assistance in Bissau, Guinea Bissau in 1987. All other medical schools appeared much later (Table 1).

Seven of the respondent medical schools were public and two, one in Mozambique and another in Guinea Bissau, were privately owned (Table 1).

### Programmes offered

Besides the medical degree offered by all the medical faculties, some offered other undergraduate (n = 4; 1 in Angola and 3 in Mozambique) and postgraduate programmes (n = 6; 2 in Angola and 4 in Mozambique) (Table 2).

**Table 2:** **Undergraduate and postgraduate health programmes in the faculties of Angola, Guinea Bissau and Mozambique**

University and faculty/unit	Country	Undergraduate programme besides medicine	Postgraduate programme
Agostinho Neto - Faculty of Medicine	Angola	No. An attempt to offer dental training in 1980/81 failed as none of the students succeeded in completing successfully the first year of training	Masters in Field and Laboratory Epidemiology
			Masters in Public Health (collaboration with the Faculty of Economics)
			Postgraduate studies in Neuro-psychology
Agostinho Neto - CEDUMED		No	Specialization course in health management (option: hospital administration)
			Master in Medical Education
Jean Piaget^a^		Clinical Psychology	No
		Pharmacy	
		Dentistry	
		Nursing and Midwifery	
		Physiotherapy	
		One year preparatory programme for prospective health students	
Instituto Superior Técnico Militar		No	Offers a Masters programme in partnership with Beira Interior University, Portugal
Katiavala Bwila (Benguela)		No	No
11 de Novembro (Cabinda)		No	No
Lueji A’Nkonde (Malange)^b^		No	No
José Eduardo dos Santos (Huambo)		No	No
Mandume ya Ndemufayo (Lubango)^b^		No	No
ISCM-CH Universidad de La Habana - Raul Diaz Arguelles	Bissau, Guinea Bissau	No	No
Jean Piaget		No	No
UniZambeze	Mozambique	Dentistry	
		Pharmacy	
Eduardo Mondlane		No	Masters in Field Epidemiology
			Masters in Public Health
UniLurio		Dentistry	Masters in Education in Health Sciences
		Pharmacy	Masters in Tropical Medicine and International Health
		Nutrition	
		Optometry	
		Nursing	
Catolica de Moçambique		Nursing	Masters in Public Health
		Clinical and laboratory exams	
		Interdisciplinary studies on	
		HIV/AIDS and health	
		Pharmacy	
		Clinical Psychology and Social Assistance	
		Administration and Hospital Management	

^a^There are several courses in health and allied sciences but not in the Faculty of Medicine; ^b^Information retrieved for university web site on 15 January 2014.

In Angola, CEDUMED was responsible for coordinating the work that led to the Internal Assessment of the Medical Programme (2005); Institutional Assessment of UAN’s Faculty of Medicine (2009); the definition of the professional desired for Angolan Medical Doctors (2009); the curricular reform of UAN’s Faculty of Medicine (2009); the first postgraduate Course in Health Management (Hospital Administration) (2004) with the support of Nova University, Portugal; 3 editions of the Masters Programme in Medical Education with the support of Oporto University, Portugal; and the edition of *Revista Angolana de Educação Médica* (*Angolan Medical Education Journal*) (4 issues, non-indexed), among others [14] (Mario Fresta, CEDUMED Director, personal communication, December 2013). CEDUMED recently gained autonomy from the Faculty of Medicine of UAN and has developed plans to dynamize research and postgraduate, masters and PhD programmes in order to strengthen HR within Angolan faculties complying with the Angolan National Plan for Education and Training.

### Applicants and registered students

Data from responding faculties on applications and registered and graduated students per year are summarized in Table 3.

**Table 3:** **Applicants and registered first year students in medical schools per gender and ratio male/female and ratio of applicants to registered students**

Country	Origin	Applicants			Ratio male/female	Registered students			Ratio male/female	Ratio applicant/registered student
		Male	Female	Total		Male	Female	Total		
Angola (n = 3)	National	1,150	1,135	2,285	1.0	123	153	276	0.8	8.3
	Foreign	0	0	0	-	0	0	0	-	-
	Total	1,150	1,135	2,285	1.0	123	153	276	0.8	8.3
Guinea Bissau	National	25	31	56	0.8	45	40	85	1.1	-
	Foreign	1	6	7	0.2	1	6	7	0.2	-
	Total	26	37	63^a^	0.7	46	46	92	1	-
Mozambique	National	52^b^	105^b^	157^b^	0.5	58^d^	84^d^	172^d^	-	-
	Foreign	2^b^	0^b^	2^b^	2.0	3^d^	1^d^	4^d^	-	-
	Total	54^c^	105^c^	159 + 400 + 2,094	-	71^c^	85^c^	176 + 168 + 169	-	-

^a^Data from only one of the two medical schools; ^b^data from three of the four medical schools studied; ^c^data refer to only one of the schools. On the total column, data is presented for the three schools; ^d^data from the four medical schools.

+ signs mean more than 159, 400 etc.

Additional data from the most recent medical schools in Angola (Huila, Benguela, Huambo, Cabinda and Malange) showed that there were 1,878 registered students in 2013: 25.0% in the first year, 20.7% in the second year, 19.9% in the third year, 14.2% in the fourth year, 14.5% in the fifth year and 5.8% in the last year of training (Table 4).

**Table 4:** **Medical students’ registrations per year of training in five Angolan public medical schools**

Província	1st year	2nd year	3rd year	4th year	5th year	6th year	Total
Huíla	109	93	67	49	49	0	367
Benguela	65	59	56	69	57	59	365
Huambo	91	100	76	55	63	0	385
Cabinda	60	68	70	47	58	50	353
Malange	145	68	104	46	45	0	408
Total	470	388	373	266	272	109	1,878

Source: National Department of Human Resources, Ministry of Health, Angola, 30 April 2013.

In 2011 there were a total of 2,285 applicants to the medical programmes of the respondent faculties in Angola (n = 3), 63 in Guinea Bissau (n = 1) and 2,653 in Mozambique (n = 4). In that same year, a total of 276 students were registered in the medical programme in 3 of the Angolan medical schools, 92 in the 2 Guinean medical schools and 682 in the 4 Mozambican schools. The number of applicants largely outnumbered the number of vacancies - for each 2009 vacancy there were 17 applicants and in 2012 there were almost 10 candidates for each vacancy.

In Guinea Bissau and Mozambique a very small proportion of the applicants were foreigners (11% and 1%, respectively). The same pattern was observed for first year students (4% in Guinea Bissau and 2% in Mozambique) (Table 3).

In general, there were more females applying to the medical degree than males. The lowest male/female ratio of the applicants was observed among Guinea Bissau foreign applicants (0.2) and in Mozambican national applicants (0.5). The overall ratio for Angola was 0.8 registered males per registered female while in Guinea Bissau the ratio was 1.1 males per female (Table 3).

### Barriers to increased intake of students

Increasing the available lecture room infrastructures, library and laboratory facilities, having more and better paid teachers, better social and financial support for students, better conditions for clinical clerkships, Internet connections and more administrative personnel were considered essential to increase the number and quality of medical graduates.

For the Angolan schools, in order to increase the quality of graduates from the medical schools, it was considered essential to have a stable teaching workforce, recruiting more teachers, increasing the number of administrative personnel and having the adequate material and qualified personnel for laboratories. On the other hand, Angolan schools also considered as necessary more funding for research as well as clearer opportunities for career development for teachers (for example, incentives for undertaking a masters or a PhD programme).

Mozambican informers considered evaluating teachers’ performance essential to increase the quality of graduates, as well as having better qualified students entering the first year. Mozambican schools also stressed the importance of having a better qualified teaching workforce.

In Guinea Bissau, the need to increase the quality of graduates was closely linked to the improvement of infrastructures such as electrical supply, access to the Internet and improved laboratory facilities. Guinea Bissau respondents also recognized the importance of a better qualified teaching workforce.

Medical schools were asked about innovations which could help to overcome the barriers to increasing the number of graduated medical students.

Angolan schools mentioned that it was essential to have vocational orientation in secondary school. Another strategy would be training monitors from the students group that could join the teaching staff later on. Angolan schools also mentioned the need to have eLearning opportunities to support traditional learning and the need for a student’s support office.

The innovations suggested by Mozambican medical schools included the creation of night courses for medical students as well as establishing national and international partnerships with universities and other institutions. On the other hand, extension activities (that is in the community) would help overcoming barriers to increasing the number of graduates as well as the involvement of governmental authorities. According to these schools, recruiting more national teachers and implementing a system for funding students would help overcome these barriers.

No suggestions on innovations were presented by Guinean schools.

### Teaching workforce

There were 117 teachers in the 3 Angolan respondent medical schools, 410 in the 4 Mozambican schools and 47 in the 2 Guinean schools (Table 5). Except for one school in Angola and another in Guinea Bissau, male teachers were more frequent than female teachers. However, when analyzing disaggregated data on gender distribution per specialty from one Mozambican school (the only one that provided this type of data) gender differences varied greatly (for example, in microbiology, pathology or paediatrics, women outnumbered men).

**Table 5:** **Total number of teachers per school and per year and teaching workforce characteristics in % (gender, origin, full-time and educational level and available teaching positions)**

Country	Medical school	Total (N)		Gender		Origin		Full-time	Educational level				Available teaching positions	Enrolment in research activities
				M	F	National	Foreign		Less than bachelor	Bachelor	MsC	PhD		
Angola	1	41	117	-	-	-	-	-	-	-	-	-	0	100.0
	2	38		50.0	50.0	15.0	85.0	-	-	-	-	-	-	0
	3	38		42.9	57.1	22.9	77.1	74.3	0	100.0*	34.3*	2.8*	0	5.2
Mozambique	1	181	410	51.4	48.6	98.0	2.0	33.1	0	76.8	9.4	13.8	0	-
	2	69		66.7	33.3	68.1	31.9	-	-	-	-	-	42.1	5.0
	3	115		53.9	46.1	60.0	40.0	33.9	1.7	53.9	12.2	2.6	20.0	10.0
	4	45		85.0	15.0	100.0	0	-	-	-	-	-	87.0	15.0
Guinea Bissau	1	42	51	45.0	55.0	16.7	83.3	-	-	-	-	-	0	100.0
	2	9		100.0	0	78.0	22.0	-	-	-	-	-	5.0	10.0

*Teachers can hold several academic degrees.

Further data obtained from one of the non-respondent schools, the Luanda UAN’s Faculty of Medicine, identified, for March 2013, a total of 148 teachers: only 4 of these were foreigners [15], as compared with the all the other Angolan and Guinean medical schools, with more than 70% of their teaching workforce being foreign. Mozambican schools relied mostly on national teachers.

Time committed to teaching was available for the teaching staff of three of the schools (two from Mozambique and one from Angola). In Mozambican schools, the percentage of full-time teachers varied between 33.1% and 33.9%. For the Angolan school (one of the recent ones), 74.2% of the teachers were fully dedicated to the medical school.

Concerning the educational level of the teachers, there were few who had a masters or a PhD qualification. The percentage of teachers with a PhD degree ranged from 3 to 13% (data on 2 Mozambican schools, one of which was the oldest, one and one1 of the recent Angolan schools). In one of the surveyed schools who answered this question, 2% of the teachers did not even hold a bachelor degree (recent school from Mozambique). For the non-respondent Luanda Faculty of Medicine already referred to, 8.8% (n = 13) had a PhD and 16.2% (n = 24) had completed their masters training [15].

Regarding available teaching positions, while in some schools all teaching posts were already filled (n = 4: the oldest school in Mozambique, 2 of the most recent in Angola and the oldest school in Guinea Bissau), in others there were positions yet to be filled (n = 4: 3 in Mozambique and 1 in Guinea Bissau). The most extreme case was one of the Mozambican medical schools, which had 87% of the teaching positions vacant.

The percentage of the teaching workforce enrolled in research activities varied greatly within and between countries. For instance, in Angola, while in one medical school all teachers were enrolled in research activities, in another, none were. In Mozambique, the percentage varied between 5 and 15% and in Guinea Bissau between 10 and 100% (Table 4).

### Available resources

We asked medical schools about the adequacy, in terms of quantity and quality, of the available resources to students/training process.

Angolan medical schools considered classrooms, teaching laboratories, Internet, and computers to be quantitatively and qualitatively adequate to the needs. On the other hand, they considered research laboratories, skills laboratories, teleconference technology, video conference technology availability of journals, residences and connections to telemedicine/radiology not adequate in terms of quantity and quality.

Mozambican medical schools identified the limitations associated with poor connections to telemedicine/radiology and to a university hospital. Research laboratories were also considered not adequate as well as technology for teleconference and videoconference. The available computers and Internet connection were considered adequate in terms of quantity and quality.

In Guinea Bissau there were no connections for telemedicine/radiology and research laboratories, Internet for students and skills laboratory were not considered adequate in quality and quantity.

## Discussion

The PSAC are among the countries with critical human resources for health (HRH) shortage. The medical workforce is one of the most affected workforces and one of the main reasons for the deficit of medical doctors is the limited training capacity of medical schools.

Angola was the first African country to graduate medical students during a short-lived experiment in the eighteenth century [16, 17]. But it was not until 1963 that medical education was reintroduced at the same time as in Mozambique. These colonial faculties remained the only schools locally graduating medical doctors for almost four decades. Guinea Bissau, with the support of Cuba, founded its first medical school in the middle of the 1980s.

However, in the past decade the three countries have shown greater commitment in creating medical schools and training more doctors. Since 2008, several schools, public and privately owned, were created in Angola, Mozambique and Guinea Bissau, bringing the medical school/population ratio to within the recommended ratio of 1 medical school for every 2 million inhabitants [18] for Mozambique (population in 2011 was 23,930,000 inhabitants, 1 medical school per 4.7 million inhabitants) [19] and Angola (population in 2011 was 19,618,000 inhabitants, 1 medical school per 2.4 million inhabitants) [20] but to a clear excess in Guinea Bissau (population in 2011 was 1,547,000 inhabitants, 1 medical school per 773,500 inhabitants) [21].

This expansion of the medical training capacity might be explained by a combination of factors: related to the peace process after civil wars in the three countries, a greater commitment with strategic health and higher education planning, including health workforce planning in three countries [22, 23], the work that led to the 2006 World Health Report and other initiatives such as the Joint Learning Initiative, that cast light on the depletion of medical doctors around the world and especially in sub-Saharan African. The excess of candidates in relation to the number of admissions to the faculty represent also an unmet demand seen as a business opportunity, leading to the appearance of private faculties in the three study countries.

This panorama is, in fact, very similar to that found in all Sub-Saharan Africa. After the creation of the first medical school in this region, in 1918 in Dakar Senegal, the number of new medical schools only increased significantly after the colonial independence, in the 1960s and 1970s. The last 20 years have witnessed a major growth in medical schools in this region, a scenario very similar to that found in the PSAC [1].

With their current capacity the annual local production of doctors will increase from about 100 to about 500 in Angola and 300 in Mozambique from 2015 onwards. For Guinea Bissau the rate of production will probably stabilize just below 100 doctors per year.

In Angola, the national production is complemented by bilateral agreements with Cuba to admit about 800 medical students every year, increasing the yearly production to above 1,000 per annum, a situation similar to that found for the bilateral agreement between Cuba and Timor Leste [24]. Data on bilateral agreements of the other two countries are not available.

The increase in the number of medical trainees represents in itself a major challenge for medical schools, and the country’s health system. The training of these students is to be made mainly on public health services which are often fragile, lacking, with poor infrastructures and without adequate clinical tutors. On the other hand, the public health system is still the major employer of young doctors and fiscal space should be created for admitting and retaining students once they graduate; otherwise, in a few years’ time, PSAC will face problems such as unemployment or underemployment, migration or dissatisfaction of the medical workforce. The recent (2013 to 2014) medical strike in Mozambique is a clear sign of this shortcoming. It is therefore surprising that none of the medical schools studied reported interaction with the Ministry of Health with a view to ensure adequate workforce planning for the country.

Despite the obvious rise in medical schools in the three countries studied (Angola, Mozambique and Guinea Bissau), several issues still prevail and that can impair the scaling-up and skilling-up of the medical workforce: high number of applicants to low number of vacancies, low graduation rates, lack of infrastructures, poor qualified teaching workforce and dependence on foreign teachers, poor teachers’ salaries, and insufficient infrastructures, among others. The increase in the number of vacancies does not reflect an increase in the quality or quantity of infrastructures such as laboratories or information technologies as it was stated by medical schools in the three countries.

On the other hand, there is serious concern about the quality of the training offered and the resulting lack of efficiency of the programmes offered. Data from the Luanda UAN Faculty of Medicine acknowledges the poor profitability of the programme offered: from the about 100 students entering the faculty, only about half graduate in the statutory 6 years of training [25]. The rate of students moving forward to the next academic year with incomplete credits from the years behind varied between 20% in Mozambique, 12% in Angola and 5% in Guinea Bissau [26].

One of the newest medical schools in Angola, which had not yet graduated the first cohort of students, also underwent an internal evaluation process based on the experience of UAN’s Faculty of Medicine conducted in 2009. The aim of this process was to identify major weaknesses and present a series of coping strategies to address them [27].

The ratio of applicants to registered students is still very high. This represents an opportunity in terms of HRH development. The effort to increase the number of medical graduates needs only to be made in terms of having more vacancies and does not require efforts to make the medical course more attractive as sometimes happens in other health courses (for example, the case of nursing in North America) [28–30].

It is also well known and debated that in order to increase the number of medical graduates it is not sufficient to simply increase the number of vacancies. This became evident as schools mentioned the need for more qualified teachers and for better infrastructures, namely laboratories. Thus, planners should account for the training capacity of the schools, the skills and competences of the teaching workforce. As stressed by recent reports on medical education [31], students should be offered the opportunity not only to learn skills but to practice them until an acceptable level of proficiency is achieved.

Besides, it should also be kept in mind that there should be fiscal space not only to train but to later absorb the newly graduate medical doctors. Although not part of the scope of the present study, it should not be forgotten that medical students will have expectations towards their professional life and that, in the countries studied, those expectations mean to be fully employed, mainly in the public sector [26, 32, 33]. Thus, when the Ministry of Higher Education (or equivalent department) gives authorization to open more medical schools, the Ministry of Health plans the intake of new medical graduates coming out of the faculties in subsequent years.

The percentage of full-time teachers is relatively low in most of the medical schools and the number of teachers with a PhD degree was very low in all medical schools. There is a general agreement that teaching staff should be given incentives to maintain a good balance between teaching experience and clinical experience, which raises questions about the suitability of full-time versus part-time teachers [31, 34].

The discussion around full-time/part-time teachers is frequent and there is no agreement on the ideal percentage of full-time teachers in medical schools. If it is true that full-time teachers have different responsibilities (for example, research besides teaching), it is also true that maintaining practice in health services can be seen as fundamental for being updated and for not losing touch with health services’ reality [34]. In some countries, where the salaries of teachers are poor (issue mentioned by several of the schools studied) keeping another job in the health sector, and thus being a part-time teacher, can be critical for maintaining a desired income [35].

The unqualified teaching workforce was pointed out by countries as a barrier for increasing the number of graduates. Opportunities should be available to all teachers to further develop their skills through PhD programmes or masters programmes. However, sometimes supporting a teacher to undergo a PhD programme means losing that teacher for some period of time (for example, to attend a programme abroad) or to lose it forever (for example, to occupy another position elsewhere). Many of these teachers are also medical practitioners within the National Health Service and it is not always possible to obtain the necessary authorizations from the Ministry of Health. As a result, both Angola and Mozambique are investing heavily in local masters and PhD programmes: this may add to the strain of managing the faculty with limited resources and benefits from the support of foreign academic institutions, as reported from other countries in Africa [2]. This is also important to retain medical doctors in the home country [1].

To compensate for the serious understaffing, medical schools recruit foreign teachers. Some are investing in their medical students, preparing them with pedagogic skills [36] and using them as mentors for more junior colleagues [37]. In the PSAC these teachers are mostly Cubans, deployed as part of Cuba’s social, medical and scientific diplomacy [38–41], with a very high turnover rate every second year, which precludes long-term planning.

Insufficient infrastructures, specially laboratories but also Internet connections and available technologies for teleconference and videoconference were mentioned as barriers in terms of quality and quantity to increasing the number of students. Medical students from the three countries have also reported this issue in previous studies [26, 32, 33].

A 2007 study among medical students from Mozambique’s public medical faculty in Maputo [42], highlighted that expectations of getting into medical school were already associated with a migration from the periphery to the capital city, even before entering medical education. This was confirmed in posterior studies again in Mozambique, but also in Angola and Guinea Bissau [26, 32, 33]. This seems to also favor the current concentration of physicians in the capital city. The current effort for the establishment of faculties outside the capital cities of Angola and Mozambique may eventually help to motivate medical students to settle and practice outside the capital city after graduation.

The feminization of the student population is similar to findings from other African countries [26, 32, 33, 43–45]. Female medical practitioners are more likely to work in the public sector and less likely to have dual practice and to work extended hours or accept positions in isolated locations. Further, female students do have a preference for controllable lifestyle specialties like those in primary health care [46–51].

## Conclusions

Medical education is an important national investment, but the returns obtained are not as efficient as expected. Investments in high-school preparation, tutoring, and infrastructure are likely to have a significant impact on the success rate of medical schools. Special attention should be given to the socialization of students and the role model status of their teachers as many of the current students will be the teachers of the future.

Governments for PSAC have significantly invested in training to address medical shortages. However, medical schools are still struggling the give an adequate and effective response. Developing a local postgraduate training capacity for doctors might be an important strategy to help retain medical doctors in the home country.

## Abbreviations

PSAC: Portuguese Speaking African Countries
WHO: World Health Organization
HRH: Human resources for health
CEDUMED: Centre for Medical Education
UAN: University Agostinho Neto.
